# Designing an IT Ecosystem for Pregnancy Care Management Based on Pervasive Technologies

**DOI:** 10.3390/healthcare9010012

**Published:** 2020-12-24

**Authors:** Daniel Bjelica, Artur Bjelica, Marijana Despotović-Zrakić, Božidar Radenković, Dušan Barać, Marko Đogatović

**Affiliations:** 1 Faculty of Organizational Sciences, University of Belgrade, 11000 Belgrade, Serbia; daniel.bjelica@gmail.com (D.B.); boza@elab.rs (B.R.); dusan@elab.rs (D.B.); 2 Faculty of Medicine, University of Novi Sad, 21000 Novi Sad, Serbia; artur.bjelica@mf.uns.ac.rs; 3 Department of Obstetrics and Gynecology, University of Novi Sad, Clinical Center of Vojvodina, 21000 Novi Sad, Serbia; 4 Faculty of Transport and Traffic Engineering, University of Belgrade, 11000 Belgrade, Serbia; m.djogatovic@sf.bg.ac.rs

**Keywords:** IT ecosystem, pregnancy care, e-health, smart healthcare, mobile technologies, ontologies

## Abstract

Pregnancy care is a topic of interest for both academia and practitioners. Novel pervasive technologies and applications, such as mobile technologies, wearables and IoT, open a wide corpus of possibilities for fostering pregnancy care management, and reducing risks and problems, improving communication among stakeholders and society development. This article introduces a model of a pregnancy care IT ecosystem based on the integration of various services in a semantically enriched e-health ecosystem. As proof of concept, both the web and mobile applications that aim to help pregnant women and gynaecologists were designed and employed in a real environment. An evaluation of the developed ecosystem was performed on a sample of 500 pregnant women and 100 doctors. After pilot usage, a survey was used to collect the data from participants, and assess the acceptance of the developed system. Results show that quality, usability and usefulness are on a high level, and that both pregnant women and doctors are ready for more extensive use of the system. In addition, research findings imply that employing pervasive technologies could bring significant benefits to all the parties in pregnancy care systems.

## 1. Introduction

Pregnancy care management is a topic of great importance to both academia and practitioners. Despite advances in healthcare, the most common implications of suboptimal pregnancy care, such as preterm birth rates, foetal development obstacles, issues with pregnant women’s mental and physical health, hypertensive complications, etc., are still widely present [1,2]. Omnipresent technologies have disruptively changed approaches and methods in this context. Technologies such as Bluetooth, RFID, NFC and IoT (Internet of Things) have pushed a variety of processes and communications within pregnancy care out of hospitals, making them ubiquitous and available to future mothers who primarily consume digital sources of information [3]. Studies have pointed out that women in prenatal care use the web, and mobile and social networks on a daily basis [4], which improves their adaptation to pregnancy [5]. At the same time, certain obstacles and issues when using digital information sources, services and applications appear: not consulting doctors, self-medication, data privacy, low digital literacy, etc. [6].

Applications of IoT, wearable and mobile technologies in pregnancy care have been studied extensively [7,8,9]. Most of them have focused on a single use-case, without a wider consideration of integration with the ecosystem of smart health care [10]. Additionally, the existing works focus on high-end products, which may be expensive or not widely available [11,12].

Having this in mind, the main goal of this research is to make pregnancy care easier to manage by enhancing communication between the main stakeholders: pregnant women and doctors using the available and pervasive technologies. In order to design a smart ecosystem and enable interoperability of different pregnancy care management components, we adapted elements from several established ontologies and created a new one. Furthermore, as proof of concept, we developed two key components of the pregnancy care system: (1) a mobile application that was evaluated among both pregnant women and doctors, and (2) a web application for the management of the system. We also performed a pilot evaluation.

Further text is structured as follows. Section 2 presents related work on pregnancy care management ecosystems, focusing on the elements of electronic health records and mobile services. In Section 3, we have developed an ontology for pregnancy care management, which serves as a core concept for the integration and interoperability of the components. Section 4 presents the design and the implementation of selected components of the pregnancy care ecosystem. In Section 5, we present the results of the application of the designed services in a real context. Finally, we provide a discussion, conclusions and implications.

## 2. Related Work

Studies investigating the effectiveness of employing IT solutions in medicine have shown promising results related to healthcare services and recommendations, risk identification during pregnancy, improvement in the satisfaction of women with provided healthcare, and strong financial benefits for the healthcare system [6]. Interest in this topic is rapidly growing due to the permanent advancement of technologies and a wide corpus of application contexts [13]. The core components of interest in this article are electronic health records and mobile services, their application in pregnancy care management, as well as the possibilities of integration with other elements of e-health information systems.

### 2.1. Electronic and Personal Health Records

Electronic Health Record Systems (EHRs) contain “information on patient medical history, diagnoses, treatments, tests, and medications, among others, medical assisted systems for elderly people, and medical data over wireless body sensor networks” [14]. The main goal is to allow doctors and other medical staff to access and share information related to the particular patient. Until recently, EHRs were seen as the main vehicle to drive healthcare systems forward [15]; however, many researchers are now highlighting the vital role to be played by patients in controlling their health information and self-managing their diseases. These recent developments have positioned the Personal Health Record (PHR) at the centre of healthcare ecosystems [16]. The personal health record is defined as an “electronic, universally available, lifelong resource of health information maintained by individuals” [17]. Previous studies explored the web, mobile and USB-based PHRs in general as well as mobile PHR (mPHRs) for particular purposes such as blood donation [3,18]. Kaelber and Pan [19] reviewed the potential economic value of PHRs, identifying their possible benefits, such as remote patient monitoring, appointment scheduling, pre-encounter questionnaires, and the ability to share key information (including medication lists, symptoms and test results) [19]. However, several issues of employing PHR were identified as well: the need for patients to have some health knowledge, usability concerns, socio-cultural influences privacy and security, etc. [3,20]. Newer approaches focus on the application of blockchain for secure storage of the PHR data, while using mobile applications as the main interface [21,22].

### 2.2. Mobile Pregnancy Care Applications

The number of mobile health applications and services has been growing rapidly over the last 10 years, resulting in disruptive changes and transformations in health practices. Mobile technology appears to be promising in enhancing access and efficiency in providing healthcare [23]. Mobile health (mHealth) has great potential in addressing disruptive issues in healthcare, given the ubiquity of mobile devices around the world, as well as their omnipresence, availability, high reach and cost-effectiveness. According to Haddad et al. [6], the most common applications used in mHealth are: client education and change in behaviour; probes and diagnoses; records and management of vital events; data collection and reports; healthcare electronic charts; electronic decision-making support; interprofessional communication; work schedule and task lists of the professional; education and professional training; human resource management; supply chain management; incentives and financial transactions. A 2019 literature review evaluated the various uses of smartphones and tablet devices in many different contexts, such as: health promotion interventions, diagnosis, treatment, monitoring and management of some of the most relevant conditions, support for screening, medical education, and clinical practice [24].

There are more pregnancy apps than any other medical field [25,26]. The main reasons for using mobile apps are information about changes related to pregnancy, tracking aspects of the user’s body during pregnancy, online discussions with other pregnant women, tracking aspects of their foetus, keeping track of information about their medical appointments, test results and keeping a pregnancy journal, uploading and storing photos of themselves while pregnant, uploading and storing foetal ultrasound images and others [26]. Lee and Moon classified all applications into three main categories: ‘entertainment’, ‘pregnancy and foetal monitoring’ and ‘pregnancy information’ [26]. The first category included games, pregnancy tests and ultrasound pranks, shopping for pregnancy-related products, quizzes to test pregnancy knowledge, gender predictors, and baby name generators. The second-largest category of apps provides functions that encourage women to monitor and survey both the foetus and the pregnant body. This includes tracking weight and waist measurements, diet, water consumption, symptoms, moods, medications, cravings, energy levels, and appetite. The third category provides a range of information about pregnancy, including details about foetal development, nutrition and exercise in pregnancy and substances and behaviours that pregnant women should avoid.

Many of the pregnancy apps are connected to EHR or mobile PHR systems. The results of the mPHR research [3] showed that, among a broad spectrum of features, no application covered all expected mPHR requests. Some applications only provided information to the users, while others focused on monitoring the information provided by the users. Further, there are numerous mobile apps within the Android/Apple stores, but without a scientific basis and with a focus on providing help to pregnant women, not being aware of other components, roles and resources within the whole process. Novel mobile applications are frequently integrated with intelligent and wearable devices that can be applied for measurement, monitoring, treatment and rehabilitation of patients [27]. Studies show that only around 12% of the apps include this feature, which is crucial to acquiring accurate and correct record values [3].

The main conclusions from the literature review are:

- the area of mobile health is emerging with a wide corpus of applications and research
- the majority of the proposed solutions were applied in experimental conditions and are based on custom made systems not being a part of a comprehensive medical IT ecosystem
- mPHR has become the de facto standard in mHealth
- there is great potential for employing sensors and IoT in general
- the majority of mobile solutions do not have a scientific background
- pregnancy care is among the most interesting in the mHealth literature.

### 2.3. Research Questions

Having in mind the wide corpus of research efforts related to the application of mobile technologies in pregnancy care, this article focuses on the following research questions:

RQ1: What are the main components and layers in the IT ecosystem for pregnancy care?

RQ2: Could an ontological framework be developed and employed in pregnancy care as a means to provide interoperability of components?

RQ3: What is the expected level of adoption of the IT ecosystem for pregnancy care, from the perspectives of pregnant women and doctors?

## 3. Designing a Pregnancy Care Ontology

When doing any kind of activity in the field of medicine, the main issue is how to enable the collection, processing and management of health information. The semantic interoperability of health information is of crucial importance [28]. In order to make these processes meaningful and feasible, defining a contextual ontology has proven to be a good solution. Ontologies are seen as one of the key pillars of the semantic web since they allow the explicit organization of knowledge to develop environments with better access to specific learning [29]. In the field of health and medical care, the main use cases of ontologies include the management of genetic information, knowledge base building for medical diagnoses, search and classification of disease information, and description of nursing processes [28].

For the purpose of this work, we aimed to define an ontology adapted to the particular needs in the pregnancy care process. The ontology will be a basis for a data layer and mobile application within a pregnancy care ecosystem.

**Figure 1:**
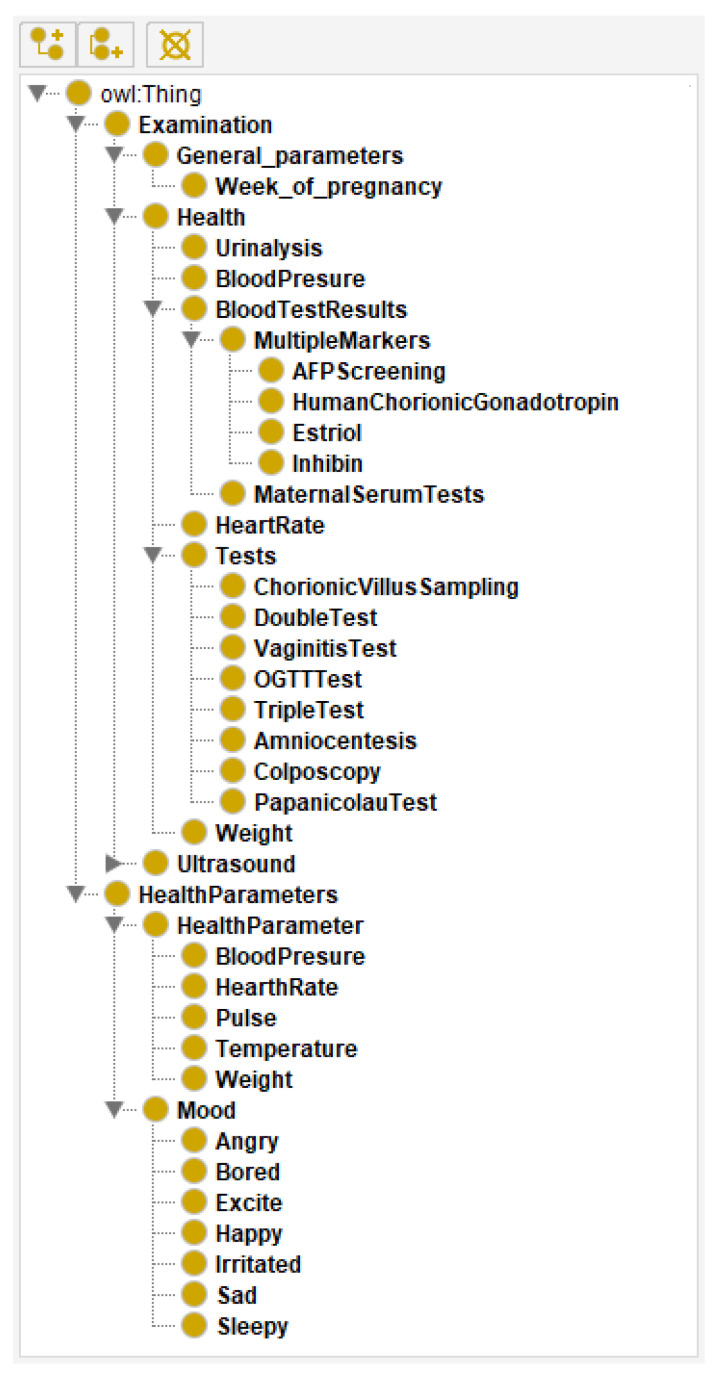
The ontology for pregnancy care.

Two ontologies were used as a starting point: Astmapp ontology [30] and SNOMED CT Pregnancy detection examination ontology [28,31]. Astmapp ontology was defined by following Methontology [32], adding a few elements to the well-known Dublin core metadata. It was the basis for the Astmapp system that “allows modelling the asthma self-management domain and reasoning over the ontology to provide trusted recommendations”. SNOMED CT ontology is a part of the Unified Medical Language System (UMLS) and aims to define clinical terms. In addition, we used OWL 2 [33] and Protégé editor [34,35]. Figure 1 portrays the main elements of the proposed ontology (screenshot from the Protege editor).

From the highest level of hierarchy there are two main classes:

- Class Examination includes all the important parts for a pregnancy check at the gynaecologist:
  - Tests—gynaecological and medical tests during pregnancy. Some of them (VaginitisTest and PapanicolauTest) are obligatory, while others depend on the pregnancy itself and imply additional checks, such as TripleTest and Amniocentesis.
  - General Parameters—parameters related to the current week of pregnancy and data about tests that should be performed.
  - Health—all important parameters that have to be tested on every check, such as blood pressure, urine analysis, heart rate and weight.
  - Ultrasound—this class includes two ultrasound checks that are performed during the pregnancy: transvaginal and abdominal.
- Class Health Parameters includes all the parameters and emotional states of the pregnant women that have to be controlled daily. These parameters include two groups:
  - Health Parameter—parameters that a pregnant woman has to pay attention to. The idea is to prevent or detect problems before they escalate.
  - Mood—a pregnant woman’s mood and mental health, such as stress, depression and other unwanted moods that could impact the foetal development.

## 4. Designing a Platform for Pregnancy Care Management

### 4.1. IT Ecosystem for Pregnancy Care Management

**Figure 2:**
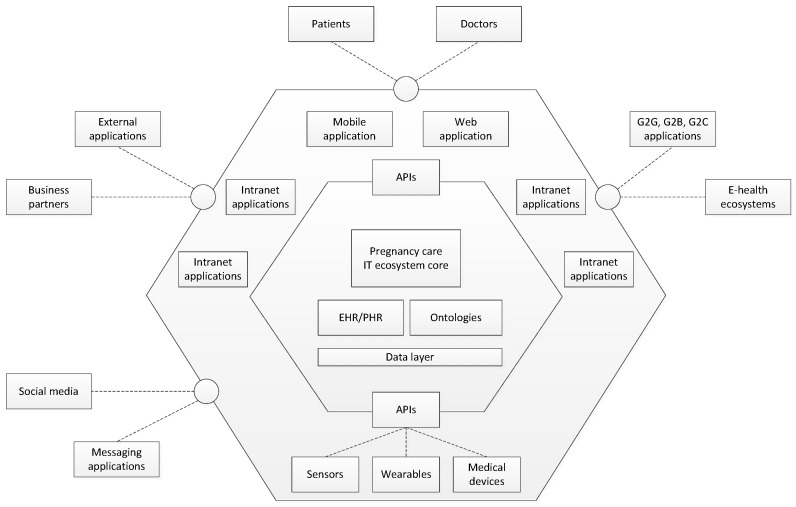
The pregnancy care ecosystem.

Figure 2 portrays a conceptual overview of an IT ecosystem for pregnancy care management. The model was created by analogy to the concept of well-established hexagonal software architecture that is used in complex software systems. The main idea of this architecture model is to create a system that is independent of external impacts and easily adaptable to the changes in any of the loosely coupled layers. The hexagonal architecture implies the separation of obligations among layers and sub-layers. Each layer has its responsibilities and defined ways of communication with other layers. Layers are connected with neighbouring layers via ports and adapters that convert messages into system procedures. The main layers on the highest level are core business processes, services and applications, and environment [36].

The main components of the proposed pregnancy care IT ecosystem are:

- Core—this is the central part of the platform that manages all the data, logic and relationships.
- Application layer—this layer includes several components:
  - Platform APIs—a corpus of REST APIs that connects the core part of the platform with all other components.
  - Mobile app—native Android application that helps pregnant women and doctors to manage pregnancy easier
  - Web application/portal/website—a common web application that enables pregnant women to manage their data related to the pregnancy as well as educational content, while the doctors are enabled to manage the whole platform data. The platform administrator manages the platform via the portal. It is also a single access point to the platform services and applications, including a lot of education content: texts, blogs, knowledge tests, FAQs, videos, forums, etc.
  - Internal applications (intranet)—the solutions that are necessary for both common and particular processes and activities: supply chain management, CRM, document management system, marketing, etc.
  - Devices and sensors—the system should be connected automatically to different kinds of devices, such as the ultrasound device. Different kinds of sensors can be integrated into the system.
- Environment—the layer that interacts with stakeholders, i.e., exchange data and managing requests from the environment. It includes integrations with external services and apps. The system needs data from other systems, web services and applications, such as calendars, disease databases, and weather services.

The core layer of the system was developed by employing PHP and NodeJS frameworks. The main principles that were followed within the implementation of the architecture were: scalability, maintainability, testability and adaptability. In order to foster content and application delivery, enhance security, facilitate availability and scalability, we opted for the Nginx web server. Further, we implemented Nginx as an API proxy to decouple the domain layer with the applications that use its APIs, and achieve higher scalability.

### 4.2. Mobile Application for Pregnancy Care Management

**Figure 3:**
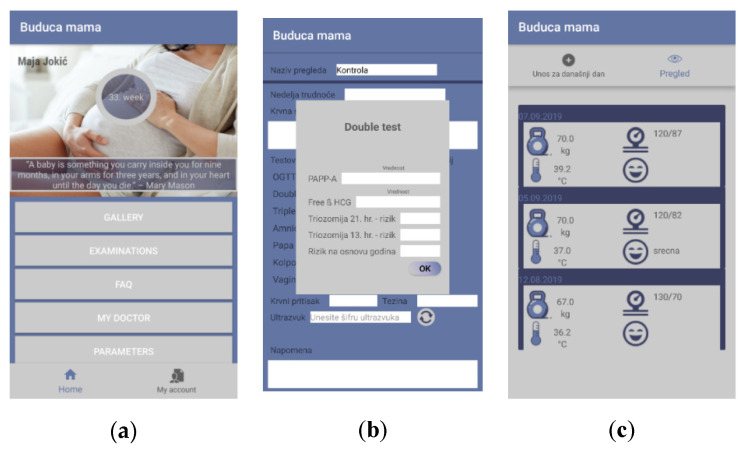
(**a**) My profile; (**b**) Double test; (**c**) Enter daily parameters.

**Figure 4:**
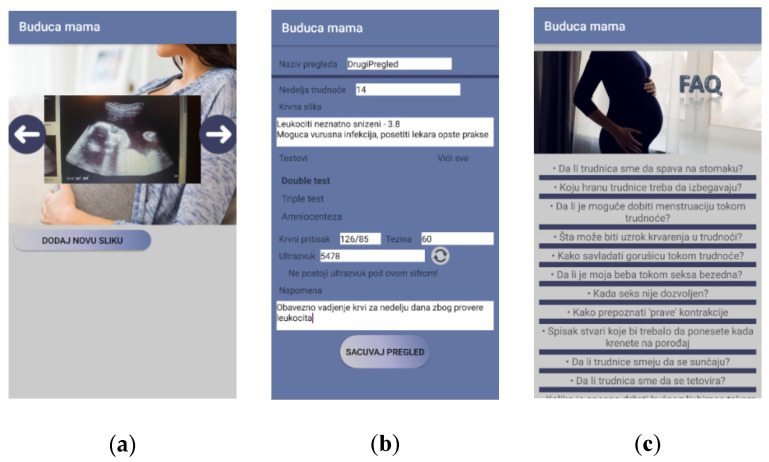
(**a**) Image from ultrasound device; (**b**) Data from ultrasound device; (**c**) FAQ.

As proof of concept, a mobile application for pregnancy care was developed. The main aim of the app is to help women to monitor their pregnancy and to enable better communication with their doctors. The app was designed so that it can easily exchange data with health applications or be fully integrated with hospital information systems. It includes a large number of parameters that were defined in the proposed ontology. The main logical units of the mobile app are:

- Mother—monitoring and entering data related to the mother herself. Further, there is a lot of educational content regarding pregnancy.
- Baby—monitoring and collecting data related to the foetus.
- Smart examination—collecting data within examinations. Data could be inserted both manually and automatically loaded from the devices/sensors.
- Doctor—the pregnant woman and the baby data management.
- The main features of the mobile application are shown in Figure 3 and Figure 4.

The developed mobile application has the following features:

- My profile—common profile page with details related to the pregnant woman
- Authentication/Registration—common features related to opening an account within the system and access to the system
- FAQ—the part that contains a vast set of information and answers that should help pregnant women
- Dashboard—a page that presents the most important data related to the pregnancy: current week, links to both checks and messages
- Doctor/Pregnant woman overview—common overview of the doctor’s/pregnant woman’s profile with basic info related to the person
- Communication—this is the common messaging system
- My checks—a list of all the recorded checks a woman had
- Check details—data related to a single check
- Image gallery—Common gallery with all the images generated through the app or via external devices within the checks
- Pregnancy parameters—this feature enables a pregnant woman to enter a set of parameters important for pregnancy, such as height, weight, mood, and blood pressure.
- Perform check—this is the page where a new check is performed, i.e., the values of the parameters are populated and saved.

Further, the mobile application includes data collected using the ultrasound device. The ultrasound device stores data in the shared cloud database, which can be accessed by the mobile application through web services and APIs. The data from the ultrasound device are available in the mobile app to both doctors and pregnant women. For the proof of concept implementation, we used the Google cloud platform Firebase for storing data and pushing notifications and messaging, but other platforms can be used as well.

### 4.3. Web Application for Pregnancy Care Management

**Figure 5:**
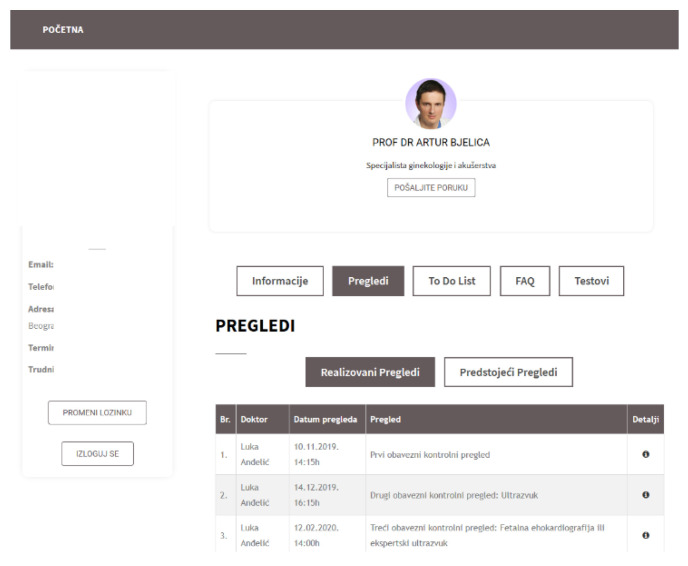
Web application—pregnant woman dashboard.

**Figure 6:**
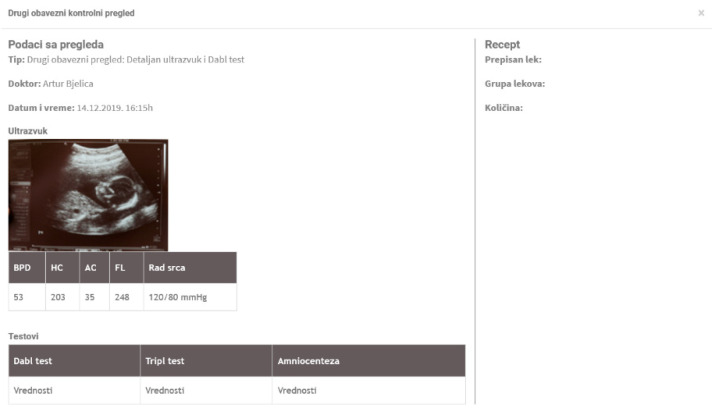
Web application—ultrasound results.

**Figure 7:**
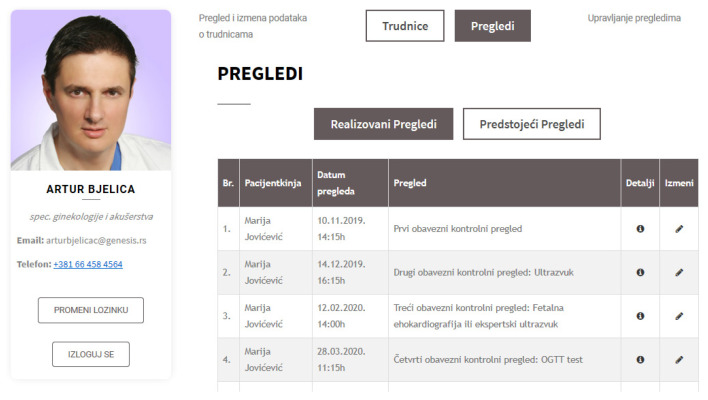
Web application—doctor’s dashboard.

Further, a web application for pregnancy care management was developed as a part of the proposed ecosystem. Figure 5 displays the main dashboard shown to a pregnant woman after she logs in to the web application. Figure 6 shows the data with ultrasound results. Figure 7 shows a doctor’s dashboard with a list of scheduled examinations. Both the web and mobile applications use the same database.

The developed IT ecosystem and its further evaluation were approved by the Ethical Committee of Hospital “Genesis” (Novi Sad, Serbia) according to the Declaration of Helsinki (Ref.No: EO 7/1/2020).

## 5. Evaluation

### 5.1. Research Settings

The aim of the evaluation of the developed system was twofold. The first goal was to investigate the potential and readiness of stakeholders for using mobile and pervasive technologies in communication, collaboration and pregnancy care. The second goal was to evaluate the mobile application developed for pregnancy care and explore its potential for further usage.

### 5.2. Data Collection and Sample

The survey sample included two groups: pregnant women (420 out of 500 returned the survey, response rate 84%) and gynaecologists (75 out of 100 returned the survey, response rate 75%). The gynaecologists were employed at nine state founded hospitals in the Republic of Serbia. The pregnant women were their patients at the time of research. They had been using the developed mobile application during pregnancy over 1–9 months. Table 1 describes the sample.

Data were collected via an online questionnaire using Google Forms during November and December 2019. Each respondent received an e-mail with a link to the questionnaire and a cover letter explaining the research and its purpose. We then contacted the non-responding patients via SMS, messaging app and social networks. The data collection process lasted for 9 weeks in total. Because empirical data were collected using questionnaires, we examined the following data collection issues: missing data, suspicious response patterns (straight-lining or inconsistent answers), outliers and data distribution. Missing data and accordingly potential non-response bias were not the issue because there was less than 1 percent of missing values per indicator. No suspicious response patterns were detected. In total, 29 incomplete questionnaires were removed.

### 5.3. Measurement Development

There were two questionnaires used in the research. The first one was created for pregnant women. It contained 21 questions of which 13 were answered using the five-point Likert scale. The second questionnaire was made for gynaecologists. It included 22 questions, of which 16 were answered using the five-point Likert scale. Both questionnaires were divided into two parts: (1) readiness and potential of mobile technologies and (2) acceptance of the application [36]. The last question was an open-ended question for suggestions on the improvement of the application. Variables used in the study were measured using multi-item scales adapted from the literature and past research and experience of the authors [1,5,37]. Table 2 shows the items we used in the questionnaire.

In addition, two questions within both questionnaires aimed to investigate what kind of mobile services respondents use for communications with patients/doctors, and in general.

### 5.4. Research Results

Table 2 shows responses regarding the level of readiness for (1) using mobile technologies and (2) services and acceptance of the developed mobile application.

In general, results for both sample groups are quite positive regarding readiness for using mobile technologies and mobile applications. Mobile devices are used quite frequently 4.45 (pregnant women) and 4.55 (doctors). Accordingly, both doctors and pregnant women strongly agree that health institutions should use mobile services for their patients (4.75 and 4.69 respectively). At the same time, the standard deviation of 1.34 within women respondents points out that there is quite a significant variation in opinions on this topic. Interestingly, the majority of respondents are familiar with the concept of pervasive technologies and different sensors for measuring body parameters.

Results related to the adjusted TAM questions prove that the quality, usability and usefulness of the developed mobile app are on a rather high level. The majority of answers are above 4.6 with a low deviation (less than 0.7). However, women’s willingness to use and recommend the application in the future is 4.2, with a deviation 0.94, which is significantly lower than other answers. This answer is somehow related to the question about the willingness for a new app installation.

An important result is related to the positive attitude of doctors who highly agree that the app improves existing PHR and includes all the important information.

**Figure 8:**
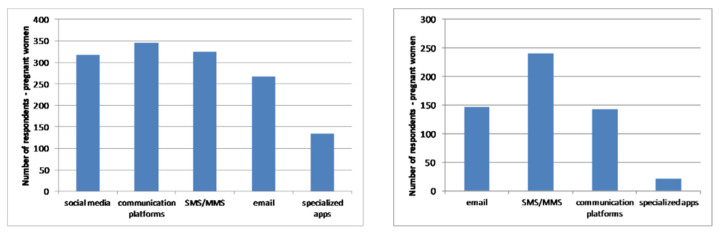
Frequency of pregnant women’ usage of mobile services (**left**) and affinity for mobile communication with doctors (**right**).

**Figure 9:**
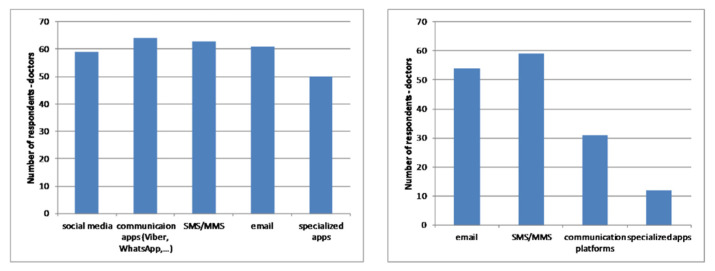
Frequency of doctors’ usage of mobile services (**left**) and affinity for mobile communication with pregnant women (**right**).

Figure 8 and Figure 9 present the frequency of using mobile technologies and services as well as an affinity toward different types of communication channels for patients (Figure 8) and doctors (Figure 9).

The level of using common mobile communication channels such as SMS, messaging applications, mobile social networks and email is quite high, as expected. However, the usage of specialised mobile applications is at a low level. Accordingly, there is a lot of space for that kind of application, which would include all common mobile communication tools and channels.

In the questionnaire, the participants were able to give their suggestions on improving the mobile application. Some of the impressions are given below.

Pregnant women:

- “It would be good if the application could enable communication about gynaecological issues other than just pregnancy”
- “Expand the list of frequently asked questions—include some topics around the birth itself and the period after it”
- “Make scheduling checks easier”
- “The application would help a lot and provide answers to questions where gynaecologists would not have to bother with regular questions. I think that this would calm pregnant women but also relieve doctors. However, I still prefer meeting with the doctor in person. “
- “Add Reminders for scheduled checks”

Doctors:

- “Expand the list of frequently asked questions—to include some topics around the birth itself and the period after it”
- “Ensure access to all pregnant women”
- “Make scheduling controls easier”
- “Emergency contact alarm when a patient has an emergency so that she can immediately receive a referral for further steps”

Regarding the usage of both mobile and web applications, it was obvious that the mobile app was consumed significantly more than the web app (89% vs. 11%). The most used features of the mobile app among pregnant women were (sorted by frequency):

- Dashboard page (initial page after logging in),
- Entering pregnancy parameters,
- Messaging,
- FAQ,
- Check details,

while the doctors mostly used: pregnant woman data preview, messaging, performing a check, and check details.

The mobile app was used on regular basis (more than once a week) by 58% of pregnant women. The majority of them (72%) used the app every day. This is influenced by the fact that they were requested to enter several pregnancy-related parameters on a daily basis. Further, the typical flow was Dashboard page->Enter health parameters. As expected, messaging and check details were used close to the dates of checks.

## 6. Discussion

Research results point out that harnessing mobile technologies could bring significant benefits to all the parties in pregnancy care systems [8,38,39]. This research aimed to investigate pregnancy care management using the holistic approach, from several perspectives: scientific, management, technologies, services and different stakeholders. Accordingly, the model of the pregnancy care ecosystem and appropriate ontology were introduced and explained.

The results related to readiness and potential of mobile technologies were extremely high (almost all the rates were above 4.7 on the scale of 1–5), which proves the well-known truth—e-health has to go mobile rapidly [23]. Previous research conforms with these, particularly that related to the role and importance of mobile PHR [18].

The main conclusions and implications of this research are related to the model of the IT ecosystem, the developed mobile app, and the developed ontology.

Model. The idea was to define a comprehensive model that would include all important aspects when designing, developing and implementing pregnancy care. Although there is a lot of research and applications in this area, very few of them discuss the issue from the system management point of view, where not only services are provided to pregnant women, but the solution is a result of system modelling. The model we proposed tries to give a solution that is highly flexible and adaptable to the changes and impact of any stakeholder. Furthermore, the model is extendable and general, so that it could be employed in any other medical context, and easily become a part of a wider smart healthcare system. Introducing good practice and a well-established form of software architecture and e-business approach to the pregnancy care system is an original contribution of the paper. As a proof of concept two components of the model were implemented and integrated.

Mobile app. Users’ feedback on the mobile app was quite positive with highly-rated features, ease of use, UI/UX, etc. Many of the existing solutions [1,2,23,40] were applied in experimental conditions and are based on closed commercial or custom made systems, which are not designed to be a part of a wider comprehensive medical IT ecosystem. The main advantage of the proposed app is that it is based on the well-established data model/ontology and integrates a comprehensive set of features found in the literature about pregnancy care apps [6]. Taking into account the limitations of employing various sensors and IoT components as well as the importance of ultrasound devices, we enabled automatic synchronization among ultrasound and PHR data. Considering the fact that the research was conducted in a developing country, results regarding readiness and familiarity with the IoT is quite positively surprising. This further supports the need for introducing sensors and smart environments in pregnancy care processes [9].

Taking into account both other research and quantitative and qualitative results of this research, the mobile app for pregnancy care should integrate common mobile communication channels, such as SMS, messaging apps, email and social networks. Making the app without full integration of these channels does not have a big chance for success. However, results regarding the lower frequency of mobile application downloads and installation should be thoroughly examined in the future. It might be a signal for health institutions to promote all the pros of the mobile applications in a better way, i.e., pregnant women will not install the apps if not asked by their doctors. In addition, this result shows that regular checks at the doctors’ should not be cut down.

Ontology. The mobile app was developed on top of the proposed ontology. Having a semantic-based approach in designing and collecting data is of high importance in IT ecosystems [30]. The developed ontology was adapted to pregnancy care and it can be used in any other system or component that deals with pregnancy, particularly those that use mPHRs and check. Research results proved that ontology was properly defined (all important parameters of a gynaecological test are included within the app 4.74 and the application could improve PHR to a great extent, 4.86). As one of the most important checks, ultrasound parameters were particularly analyzed and added to the ontology. This gives further potential for implementing advanced services based on artificial intelligence and machine learning.

## 7. Conclusions and Future Work

Pregnancy care is a topic of great interest to both academia and practitioners. Novel pervasive technologies and applications open a wide corpus of possibilities for fostering pregnancy care management, reducing risks and problems, improving communication among stakeholders and society development. The main scientific contribution of the research is the development of a model of a pregnancy care IT ecosystem based on adjusted ontology. Further, several components of the pregnancy care ecosystem were developed and implemented. The research showed the potential for using mobile applications and technologies for pregnancy care processes for both pregnant women and doctors.

### 7.1. Limitations

The research has certain limitations as a result of the fact that this was the first phase of the pregnancy care IT ecosystem implementation. Although the presented app showed great potential for use in pregnancy care, several components are not available off-the-shelf and require some time to be integrated within the system. The level of integration with other system components is still low and digital transformation is not fully achieved. As the respondents stated, the application itself should provide a few more important features, such as scheduling, reminders, improved FAQ section, integration with social channels, and emergency alerts. Further analysis of ontology’s value in practice is required as well. Additionally, the evaluation in this phase was oriented toward general acceptance of the proposed system, so the data on acceptance among certain demographic cohorts was not studied. Further evaluation will also have to address the limitations regarding the used questionnaire, through piloting the developed questionnaires, addressing the consistency and reliability issues with formal statistical methods, and considering the potential biases.

### 7.2. Future Work

Future research is primarily directed at introducing more IoT components in the system, particularly using specific sensors that would lead to gathering and controlling more data in real-time. Further, the idea is to implement an omnichannel strategy in the sense of providing data about patients from different sources, such as web applications, social networks, and emails. This would lead to extending data available in PHR and implementing the customer data platform concept. In addition, we are working on a smart recommendation system that would help both pregnant women and doctors to manage pregnancy [30]. To achieve this, it will be necessary to perform a wider evaluation of the proposed system, in order to identify information and services suitable for different cohorts of pregnant women. Further evaluation should also point out potential barriers to acceptance of the system. Finally, all the stakeholders in the ecosystem should try to disseminate results and make a broader audience aware of the potentials of the presented approach and technologies.

## Figures and Tables

**Table 1:** Demographic information related to the sample.

Measure	Item	No.	%
Pregnant women (420)			
Age	≤25	64	15.23
	25–35	196	46.67
	36+	160	38.1
Pregnancy (order number)	First	144	34.29
	Second	133	31.67
	Third	109	25.95
	Forth	34	8.1
Educational level	Secondary school	114	27.14
	Vocational school	89	21.19
	University degree	217	51.67
Gynaecologists (75)			
Sex	Female	45	60
	Male	30	40
Age	≤40	30	40
	41–50	24	32
	51–60	14	18.67
	61+	7	9.33
Years of work experience in gynaecology	0–10	23	30.67
	11–20	34	45.33
	21+	18	24

**Table 2:** Results of the research.

Indicator	Mean	SD	CI (Alpha = 0.05)
Readiness and potential of mobile technologies—pregnant women/doctors			
How frequently do you use mobile technologies	4.41	0.658	0.421
	4.55	0.53	1.029
How frequently do you install mobile applications	3.495	1.253	0.334
	3.707	0.983	0.839
I am informed about the IoT concept and omnipresent technologies for measuring body parameters	4.1	1.34	0.392
	4.373	0.97	0.99
Health institutions should use mobile services and applications for communication with patients	4.795	0.611	0.456
	4.65	0.73	1.095
Acceptance of the application—pregnant women/doctors (adjusted TAM questions)			
The applications is useful	4.761	0.634	0.455
	4.667	0.664	1.056
I would use the application again	4.24	0.938	0.405
	4.547	0.759	1.03
I think the application improves communication between pregnant woman and gynaecologist	4.795	0.65	0.459
	4.84	0.494	1.095
It is easy to find information and options within the application	4.7	0.674	0.45
	4.787	0.552	1.083
The application allows me to follow data related to the checks in an easier way	4.681	0.802	0.416
	4.773	0.534	1.08
The applications structures data related to the checks in the proper way	4.642	0.709	0.444
	4.707	0.564	1.065
IoT component would improve the application to a great extent	4.662	0.754	0.433
	4.707	0.653	1.065
The application’s flow suits me	4.536	0.909	0.434
	4.733	0.577	1.071
I would recommend the app to other pregnant women	4.786	0.608	0.458
	4.787	0.527	1.083
The application makes pregnancy care easier	4.662	0.754	0.446
	4.787	0.501	1.083
The app enables faster and more efficient treatment of the patients	4.758	0.640	0.455
	4.733333	0.578	1.083
All the important parameters from the checks are included within the application	/	/	/
	4.747	0.572	1.074
The application improves existing PHR	/	/	/
	4.867	0.476	1.101

## Data Availability

The data presented in this study are available on request from the corresponding author.
